# Effectiveness and tolerability of chlormethine gel for the management of mycosis fungoides: a multicenter real-life evaluation

**DOI:** 10.3389/fonc.2023.1298296

**Published:** 2024-01-04

**Authors:** Silvia Alberti-Violetti, Marco Ardigò, Cesare Massone, Alessandro Pileri, Raffaella Sala, Miriam Teoli, Vieri Grandi, Pietro Quaglino, Nicola Pimpinelli, Emilio Berti

**Affiliations:** ^1^ Dermatology Unit, Fondazione IRCCS Ca’ Granda Ospedale Maggiore Policlinico, Department of Pathophysiology and Transplantation, University of Milan, Milan, Italy; ^2^ San Gallicano Dermatological Institute IRCCS, Rome, Italy; ^3^ Dermatology Unit & Scientific Directorate, Galliera Hospital, Genova, Italy; ^4^ Dermatology, IRCCS Azienda Ospedaliera-Universitaria di Bologna, Policlinico di Sant’Orsola, Bologna, Italy; ^5^ Department of Medical and Surgical Sciences, Alma Mater Studiorum University of Bologna, Bologna, Italy; ^6^ Dermatology Unit, ASST Spedali Civili and University of Brescia, Brescia, Italy; ^7^ Department of Health Sciences, Dermatology Unit, University of Florence, Florence, Italy; ^8^ Department of Medical Sciences, Dermatology Clinic, University of Turin, Turin, Italy

**Keywords:** chlormethine gel, CL gel, mycosis fungoides, MF, cutaneous T-cell lymphoma

## Abstract

**Background:**

Topical chlormethine (CL) is recommended as a first-line treatment for early-stage mycosis fungoides (MF) and in 2017, the European Medicines Agency approved the CL gel formulation to treat adult patients. More recently, to increase patient compliance and adherence, clinicians have developed flexible protocols that allow the concomitant use of CL gel with topical corticosteroids in daily practice regimens. Therefore, sharing real-life data on CL gel use and side effects management may help improve the use of this agent.

**Objectives:**

To expand knowledge about the actual use of CL gel in patients with MF, the present study assessed the improvement of MF skin lesions after CL gel treatment and provided information on the management of cutaneous adverse events (AEs) in a real-life setting.

**Methods:**

This was an Italian retrospective study conducted among six dermatology referral centers. Patients ≥18 years affected by MF and in treatment with CL gel (160 µ/g), alone or in combination according to routine clinical practice, between December 2019 and December 2021 were considered. The study’s primary aim was to evaluate the effectiveness of CL gel in terms of overall response rate (ORR) after 3 months of treatment.

**Results:**

A total of 79 patients (61% male) with different stages of MF (84% early stage) were included. CL gel was prescribed mainly in association with topical corticosteroids (66% of patients). ORR after 3 months of treatment was 42%, with no differences between early- and advanced-stage MF. Response rates improved over time up to 97% after 18 months of treatment. Overall, 66 AEs were reported in 67% of patients; most were hyperpigmentation (45%) and irritant contact dermatitis (37%). Six AEs led to treatment discontinuation, and five out of six (83%) patients who reported these events resumed treatment after interruption. No AEs were classified as severe.

**Conclusions:**

Our observations support the use of CL gel in patients with early- and advanced-stage MF, making it a valuable treatment option.

## 1 Introduction

Among cutaneous T-cell lymphoma, the most common form is represented by mycosis fungoides (MF), involving about 60% of cases (1, 2). Skin malignant T-cell infiltration represents the main characteristic of MF, which initial presentation generally involves skin patches and/or plaques which can progress to tumors or erythroderma (3, 4). Diagnosis is frequently delayed and made after several biopsies, and the staging is based on the 2007 TNMB revision (5–7).

Guidelines for the treatment of MF are based on the stage of the disease and primarily aim to reduce symptoms, prevent disease progression, and improve the quality of life (QoL) (8). Currently, topical chlormethine (CL), an alkylating agent used successfully in the treatment of cutaneous T-cell lymphoma since the 1950s, represents the first-line treatment for early-stage patients, according to recommendations by the National Comprehensive Cancer Network and international guidelines (1, 9–11). CL acts by a cytotoxic mechanism on DNA, predominantly altering the neoplastic cell growth and increasing the immunogenic potential of the host (12, 13).

Initially, CL was formulated in an aqueous solution, whose use was limited by the high rate of skin hypersensitivity. The formulation of CL as a vaseline-based ointment partially improved the tolerability, but the rapid drug degradation limited the effectiveness of this formulation (14). In 2013, a multicenter phase II, randomized, blinded study (201 study) compared the efficacy and safety of 0.02% CL ointment with 0.02% CL gel in patients with persistent or recurrent stage I or IIA MF for whom corticosteroids were forbidden, demonstrating the non-inferiority of the gel (15). In this study, the authors recorded longer and faster responses in patients treated with gel compared with the ointment treatment (15). As in previous literature evidence, no detectable systemic absorption of the drug was reported (15, 16). The gel formulation was approved by the European Medicines Agency in 2017 for treating adult patients with MF, and is now commercially available in several European countries (17). In Italy, CL gel is indicated for the treatment of MF in all stages.

More recently, to increase patient compliance and adherence, and prevent treatment discontinuation, clinicians have developed more flexible protocols that allow also the concomitant use of CL gel with topical corticosteroids in daily practice regimens (18–20). Therefore, sharing real-life data on actual use of CL gel and side effect management in clinical practice may help improve the use of this agent.

In the present study, we assess the improvement of skin lesions after CL gel treatment, alone or in combination, as well as information on managing cutaneous adverse events (AEs) according to daily clinical practice. We aim to expand knowledge about the use of CL gel for MF, specifically to determine whether the clinical benefit of topical CL gel persists during long-term therapy, and to evaluate local AEs.

## 2 Patients and methods

### 2.1 Study design and patients

This was an Italian retrospective study conducted among six dermatology referral centers (Milan, Rome, Genoa, Bologna, Brescia, and Florence) belonging to the Italian Lymphoma Foundation (“Fondazione Italiana Linfomi, FIL”) cutaneous lymphomas task force. Adult patients with MF and treated with CL gel (160 µg/g), alone or in combination according to routine clinical practice, between December 2019 and December 2021 were considered. Patients for whom retrospective data or information on the type and duration of therapy and clinical outcomes were not available were excluded. The study was conducted according to the Good Clinical Practice International Conference on Harmonization (GCP ICH) E6 following the ethical principles of the Declaration of Helsinki and current legislation on non-profit observational studies. The study protocol was notified to the Ethics Committee of Milano Area 2 (protocol number 7202; 10/02/2022). All the participants signed an informed consent form.

### 2.2 Study measures

For each patient, the following data were retrieved and collected in a dedicated and anonymized database: gender, age, ethnicity, concomitant therapies, diagnosis (clinical variants), stage, modified severity-weighted assessment tool (15) at the start of therapy and after 3, 6, 12 and 18 months (where applicable) of therapy. AEs and possible disease progression were also reported.

The study’s primary aim was to assess the effectiveness of CL gel in terms of overall response rate (ORR), intended as the percentage of patients who achieved a complete (CR) or partial (PR) response to the treatment after 3 months of treatment. The definition of CR and PR was based on the EORTC-ISCL-USCLC (European Organization for the Research and Treatment of Cancer, International Society for Cutaneous Lymphomas, United States Cutaneous Lymphoma Consortium) criteria (21).

The secondary objective was to compare ORR at 3 months according to the disease stage (early stage: IA, IIA, IB *vs* advanced stage: IIB). We also seek to report the ORR after 6, 12, and 18 months of treatment according to the stage of disease (early-stage *vs* advanced-stage), based on proposed treatment duration according to clinical practice, and managed according to the response to therapy. AEs were collected including the type of skin reaction (allergic contact dermatitis assessed through patch test or irritant contact dermatitis) and the percentage of patients who continued the drug application after suspension due to AEs. Correlation analyses were also performed to assess the relationship between skin reactions and ORR.

### 2.3 Statistical analysis

Continuous variables were shown as mean and standard deviation (st.d) or median and interquartile range (IQR) and categorical variables as number and percentage. The Chi-square test was used to compare categorical variables. ORR time was estimated using the Kaplan-Meier method. Odds ratios (OR) were reported with 95% CIs.

Patients with available data at baseline and relevant time points were included. This results in a diverse population dimension (n) for each parameter at each time point, characteristic of a real-world study.

## 3 Results

A total of 79 patients (n=48, 61% male) with different stages of MF (n=66, 84% early stage) were included in the study. The median age was 63 years (IQR: 52.3–70). Most patients had classical MF (n=60, 76%).

CL gel administration consisted of daily or alternate application, depending on the skin reaction. CL gel was administered with TCS in 66% of patients (n=52). The most used were high-potency topical steroids. Most patients followed an alternate application of CL (4–5 days a week; n=49; 64%). The percentage of patients who used additional TCS was significantly higher in the alternate CL application group (n=40; 82%) than in the daily application group (n=12; 43%; p<0.001). The clinical characteristics and treatment regimens of the involved patients are summarized in **Table 1**.

**Table 1:** Summary of clinical characteristics and treatment regimens.

Characteristics (n=79)	n (%)
Male	48 (61)
Age (years), median (IQR)	63 (52.3-70)
Caucasian	79(100)
Stage	
IA	38 (48)
IB	18 (23)
IIA	10 (13)
IIB	13 (16)
Diagnosis	
Classical MF	60 (75)
Folliculotropic MF	15 (19)
Pagetoid reticulosis	2 (3)
Poikilodermic MF	2 (3)
Treatment regimens*	
Monotherapy	25 (31)
Combination with TCS	52 (66)
Missing data	2 (3)
Frequency*	
Daily	29 (37)
Alternate (4–5 days/week)	50 (63)

*n=77.

MF, Mycosis fungoides; st.d: standard deviation; TCS, topical corticosteroids.

A total of 35 (44%) patients had concomitant therapies that started before the prescription of CL gel therapy. Among these patients, 12 were advanced stage (92% of all advanced-stage patients) and 27 early stage (41% of all early-stage patients). Concomitant therapies were mainly bexarotene (n=13, 16%), acitretin (n=9, 11%, all patients were in the early stage), and local radiotherapy (RT; n=8, 10%). **Supplementary Table 1** summarizes the concomitant therapies according to the disease stage.

### 3.1 Effectiveness of CL gel treatment

ORR was 42% (n=32) after 3 months of treatment. CR was reported in 12 patients (16%), PR in 20 patients (26%), 43 patients (57%) had stable disease (SD), and one patient had a progression of disease (PD).

### 3.2 Secondary outcomes

Response rates increased over time, starting from 42% (n=32/76) after 3 months of treatment up to 97% (n=31/32) after 18 months of treatment (**Table 2**). A total of 21/32 (74%) patients responding at 3 months stopped the application before 12 months of treatment because of achieving CR.

**Table 2:** Overall response rate (complete or partial response) by stage and visit.

Stage	Early-stage disease	Advanced stage disease	Total	Chi-squared p-value
3 months	n=63	n=13	n=76	0.366
ORR	28 (44%)	4 (31%)	32 (42%)	
PD or SD	35 (56%)	9 (69%)	44 (58%)	
6 months	n=55	n=12	n=67	0.012
ORR	43 (78%)	5 (42%)	48 (72%)	
PD or SD	12 (22%)	7 (58%)	19 (28%)	
12 months	n=32	n=7	n=39	0.783
ORR	26 (81%)	6 (86%)	32 (82%)	
PD or SD	6 (19%)	1 (14%)	7 (18%)	
18 months	n=26	n=6	n=32	0.037
ORR	26 (100%)	5 (83%)	31 (97%)	
PD or SD	–	1 (17%)	1 (3%)	

ORR, overall response rate; SD: stable disease; PD, progression of disease.

After 3 months of treatment, no significant differences were observed in the proportion of patients achieving CR or PR between early-stage (n=28, 44%) and advanced-stage (n=4, 31%; Chi-squared p=0.366) disease (**Table 2**). Otherwise, after 6 and 18 months of treatment, the percentage of patients with CR or PR was significantly higher in patients with early-stage disease than in those with advanced-stage disease (6 months: n=43, 78% vs n=5, 42%, Chi-squared p=0.012; 18 months: n=26, 100% vs n=5, 83%, Chi-squared p=0.037) (**Table 2**).

**Figure 1:**
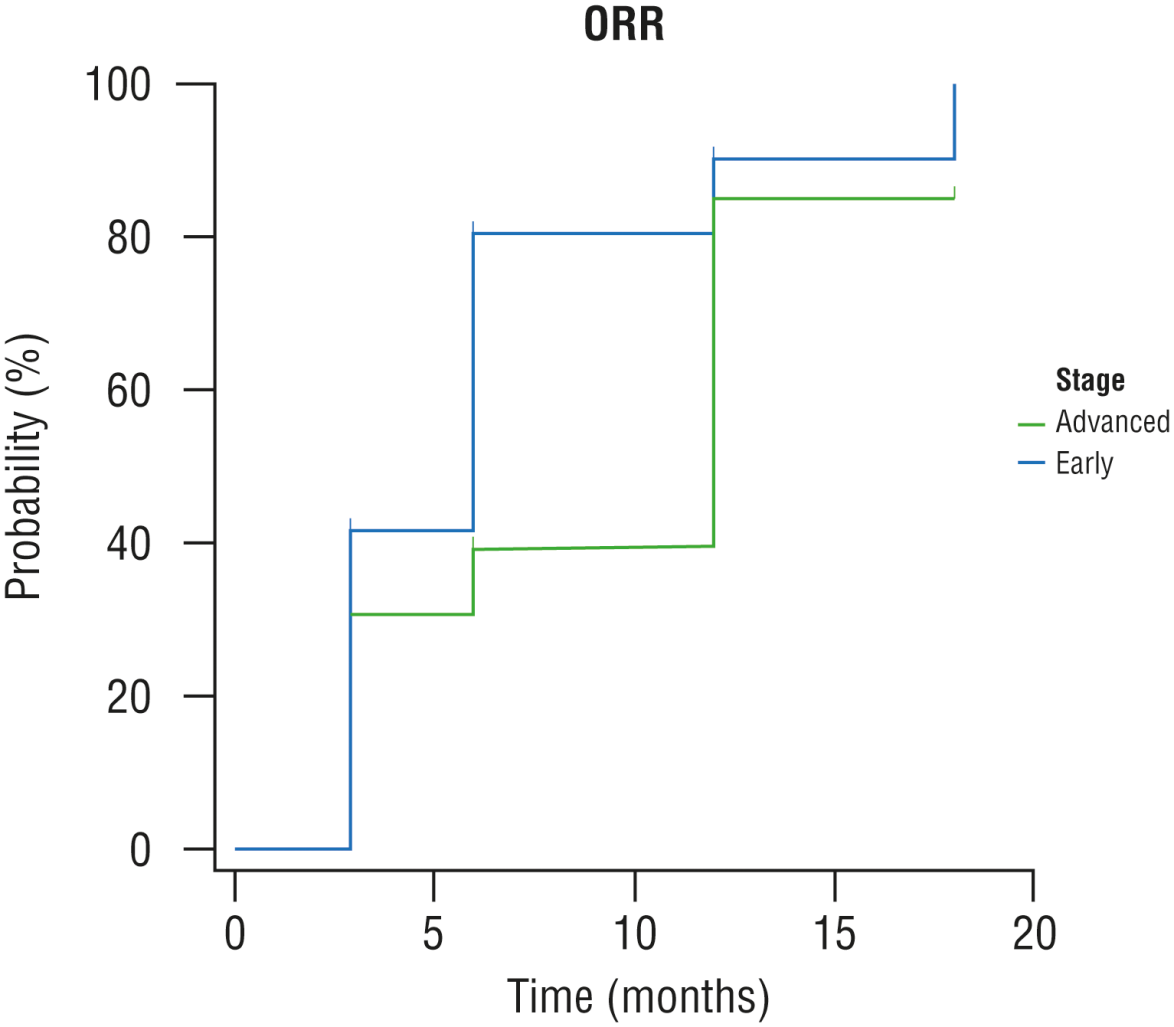
Kaplan-Meier curves, time for overall response rate by stage. ORR: overall response rate.

The mean time to CR/PR was 7.0 ± 0.6 months. The median time to response was significantly shorter in patients with the early-stage disease than those with the advanced-stage disease (6.0 vs 12.0 months; p=0.042; **Figure 1**).

The mean percentage of skin lesions treated with CL gel among patients with early-stage disease was significantly higher (87%, st.d: 18%) than in advanced-stage patients (67%, st.d: 31%; p=0.002). In the same group of patients, a trend toward the absence of PD was reported (n=55, 85% of patients had no PD), compared with patients with advanced-stage disease (n=8, 62% of patients had no PD; Chi-squared p=0.056). At the same time, a trend toward a lower percentage of treated lesions was reported in patients with PD compared with patients who did not have a progression of disease (85%, st.d: 20% vs 77%, st.d: 20%; p=0.214).

### 3.3 Safety

The median treatment duration was 26 weeks (1–120 weeks). Overall, 66 AEs were reported in 53 patients (67%); most AEs were hyperpigmentation (n=36, 45%), followed by irritant contact dermatitis (ICD; n=29, 37%) (**Table 3**). TCS were prescribed to 28 out of 29 (97%) patients who experienced ICD. The frequency of ICD was significantly higher in patients with alternate applications compared with daily application (46% vs 21%, OR: 0.3, CI: 0.1–0.9, p=0.028) (**Table 3**). The frequency of hyperpigmentation was lower in patients with alternate applications, but the statistical significance was not reached (58% vs 38%, p=0.141) (**Table 3**).

**Table 3:** Adverse events by the number of applications.

Adverse events	Daily application (n=29)	Alternate application (n=50)	Total (n=79)
ACD, n of events (%)	–	1 (2)	1 (2)
ICD, n of events (%)	6 (21)	23 (46)	29 (37)
Hyperpigmentation, n of events (%)	17 (58)	19 (38)	36 (45)
Total AEs, n of events (%)	23 (35)	43 (65)	66 (100)

ACD, Allergic contact dermatitis; AE, Adverse event; ICD, Irritant contact dermatitis.

Six (19%) AEs led to treatment discontinuation, and five out of six (83%) patients who reported these events resumed treatment after a period of interruption. No AEs were classified as severe.

#### 3.3.1 Correlation analyses

Considering the best response, a significantly higher ORR was reported in patients with hyperpigmentation (94%) than in patients without hyperpigmentation (77%; p=0.034; **Supplementary Table 2**). No correlation was observed between the occurrence of ICD and the ORR (**Supplementary Table 3**).

## 4 Discussion

CL gel is the first skin-directed therapy purposely developed for the treatment of MF and is currently recommended as first-line therapy for treating adult patients with MF by international guidelines (1, 9, 10).

According to guidelines, skin directed therapies can be associated with systemic therapies in MF patients with advanced-stage disease, as a complement to optimize the response to these therapies and reduce side effects (22, 23). In real-life management of MF, patients with early- and advanced-stage disease are prescribed more flexible CL gel regimens than those reported in the pivotal study, providing also combined treatment with low-frequency TCS application or radiotherapy or other treatments (15, 18, 19, 24). However, with the exception of the PROVE study, real-life data on CL gel use and side effect management in clinical practice are limited mainly to small populations or case series (12, 25–30).

Our study included 79 patients, representing one of the largest patient cohorts in reference to available real-life studies. In our cohort, most patients were in the early stage (84%), and CL gel was mainly prescribed in combination with TCS (66% of patients), according to the guidelines (9–11). In 92% of cases, advanced-stage patients followed a concomitant therapy. In these patients, CL gel was mainly used on the difficult-to-treat areas, which were more resistant to systemic therapies.

Our findings showed that CL gel was effective for treating lesions in 42% of patients after 3 months of therapy, with no differences between early- and advanced-stage MF (Chi-squared p=0.366). These data align with previous evidence from real-world literature, which reports an ORR between 40–50% (25–30).

After 6 and 18 months of treatment, the percentage of patients with CR or PR was significantly higher in patients with early-stage disease than in those with advanced-stage disease (p=0.012 and p=0.037, respectively). Moreover, the mean time to clinical response was shorter in patients with early-stage disease than in those with advanced-stage disease (p=0.042). The mean percentage of treated lesions was higher in early-stage patients compared with advanced stage (p=0.002). This result could be related to the trend toward the absence of PD observed in this patient group, considering also that the percentage of treated lesions was lower in patients with PD. This observation aligns with previous real-life evidence suggesting that patients with early-stage disease may have more-durable responses or higher response rates to CL gel than patients with advanced-stage disease (28).

We observed that response rates increased over time (from 42% at 3 months to 97% at 18 months), highlighting the importance of constant treatment with CL gel, as previously observed (28, 30).

Throughout the current study, 39% of patients developed some degree of dermatitis. Among them, almost all patients (97%) who experienced ICD were also prescribed TCS. Previous prospective and real-life studies supported this combined approach and reported that the use of TCS allowed the long-term administration of CL gel until clinical response, without altering its activity (26, 31, 32). In particular, in the prospective, randomized, open-label MIDAS study, the use of topical triamcinolone ointment showed to be a useful adjuvant when treating patients with CL gel, reducing inflammation without altering the effectiveness of the therapy.^28^ Only six patients using CL gel discontinued the treatment and, in five cases, patients resumed treatment with reduced applications. Although dermatitis is commonly reported during CL gel treatment, a reduction in the frequency of treatment administration may reduce treatment discontinuation, and the occurrence of dermatitis does not affect the likelihood of response, as observed in our cohort (33). These data support the versatile use of CL gel with dose and application rate adaptability.

In our cohort, 35 patients experienced skin hyperpigmentation, which spontaneously resolved after the treatment. Moreover, the ORR was significantly higher in patients with hyperpigmentation (94%) than those without hyperpigmentation (77%; p=0.034). Consistent with previous observations, our results support the association between hyperpigmentation and post-inflammatory dermatitis in regressing lesions, with an effect on melanocytes, as evidenced by an increase in the basal number of melanocyte (27).

This analysis has some limitations typical of observational studies; for example, the reduced number of patients at follow-up, particularly at 12 and 18 months and the inaccurate monitoring of the onset of side effects. However, the assessment of the duration of response after treatment discontinuation due to CR was not among the aims of the study, which were the ORR and safety of CL gel treatment. In addition, therapeutic regimens were not-standardized, as frequently observed in real-life studies, with most of advanced-stage patients performing concomitant therapies (92%), even if the use of systemic treatments was prior to the introduction of CL gel. For completeness of information, it should be reported that at the time of the study, maintenance therapies after CR were not part of clinical practice.

Despite these limitations, our study includes one of the largest series of MF patients treated in a real-world practice setting, providing useful data to optimize the daily use of CL gel treatment.

## 5 Conclusion

This study represents one of the largest series of MF patients treated in a real-world practice setting. Results showed that after 3 months of treatment, CL gel was effective in MF patients with both early- and advanced-stage disease, with a good tolerability profile. Response rates increased over time, highlighting the importance of continued treatment with CL gel. According to our data, CL gel can be effectively and safely used in combination with other therapies for MF. In conclusion, our observations support the use of CL gel in patients with early- and advanced-stage MF, making it a valuable treatment option.

## Data availability statement

The original contributions presented in the study are included in the article/**Supplementary Material**, further inquiries can be directed to the corresponding author.

## Ethics statement

The studies involving humans were approved by Ethics Committee of Milano Area 2 (register number 7202, 10/02/2022). The studies were conducted in accordance with the local legislation and institutional requirements. The participants provided their written informed consent to participate in this study.

## Author contributions

SA-V: Conceptualization, Data curation, Formal analysis, Writing – original draft, Writing – review & editing. MA: Data curation, Formal analysis, Writing – review & editing. CM: Data curation, Formal analysis, Writing – review & editing. AP: Data curation, Formal analysis, Writing – review & editing. RS: Data curation, Formal Analysis, Writing – review & editing. MT: Data curation, Formal Analysis, Writing – review & editing. VG: Data curation, Formal Analysis, Writing – review & editing. PQ: Data curation, Formal Analysis, Writing – review & editing. NP: Data curation, Formal Analysis, Writing – review & editing. EB: Data curation, Formal Analysis, Writing – review & editing.
