# Supplementary material for: Effectiveness and tolerability of chlormethine gel for the management of mycosis fungoides: a multicenter real-life evaluation
Source: Front Oncol. 2024 Jan 4;13:1298296. doi: 10.3389/fonc.2023.1298296 (PMC10794371; doi:10.3389/fonc.2023.1298296)
Supplement: Supplementary file 1 [file Table_1.docx]

Supplementary Material

**Supplementary Table 1.** Concomitant therapies

| **Concomitant therapies n (%)** | **Early-stage disease  (n=66)** | **Advanced stage disease (n=13)** | **Total (n=79)** |
| --- | --- | --- | --- |
| Acitretin | 9 (14) | 0 | 9 (11) |
| Bexarotene | 6 (9) | 7 (54) | 13 (16) |
| RT | 2 (3) | 6 (46) | 8 (10) |
| ECP | 2 (3) | 0 | 2 (3) |
| Phototherapy | 4 (6) | 0 | 4 (5) |
| Interferon alpha | 3 (4) | 3 (23) | 6 (9) |
| Mtx | 1 (2) | 0 | 1 (1) |
| RT: radiotherapy; ECP: extracorporeal photopheresis; Mtx: methotrexate | | | |

**Supplementary Table 2.** Correlation between hyperpigmentation and clinical response.

| **Time point** | **Patients with hyperpigmentation** | **Patients without hyperpigmentation** | **p-value** |
| --- | --- | --- | --- |
| 3 months:   - CR or PR - PD or SD | n=33   - 18 (55%) - 15 (46%) | n=43   - 14 (33%) - 29 (67%) | 0.056 |
| 6 months:   - CR or PR - PD or SD | n=30   - 22 (73%) - 8 (27%) | n=37   - 26 (70%) - 11 (30%) | 0.784 |
| 12 months:   - CR or PR - PD or SD | n=16   - 12 (75%) - 4 (25%) | n=23   - 20 (87%) - 3 (13%) | 0.345 |
| 18 months:   - CR or PR - PD or SD | n=10   - 10 (100%) - 0 | n=22   - 21 (96%) - 1 (4%) | 0.500 |
| Best response:   - CR or PR - PD or SD | n=35   - 33 (94%) - 2 (6%) | n=43   - 33 (77%) - 10 (23%) | 0.034 |
| ORR; Overall response rate; CR: Complete Response; PR: Partial Response; PD: Progressive disease; SD: Stable disease | | | |

**Supplementary Table 3.** Correlation between irritant contact dermatitis and clinical response

| **Clinical response** | **Patients with ICD** | **Patients without ICD** | **p-value** |
| --- | --- | --- | --- |
| 3 months:   - CR or PR - PD or SD | n=28   - 13 (46%) - 15 (54%) | n=48   - 19 (40%) - 29 (60%) | 0.063 |
| 6 months:   - CR or PR - PD or SD | n=25   - 21 (84%) - 4 (16%) | n=42   - 27 (64%) - 15 (36%) | 0.086 |
| 12 months:   - CR or PR - PD or SD | n=12   - 10 (83%) - 2 (17%) | n=27   - 22 (82%) - 5 (19%) | 0.891 |
| 18 months:   - CR or PR - PD or SD | n=9   - 9 (100%) - 0 | n=23   - 22 (96%) - 1 (4%) | 0.532 |
| Best response:   - CR or PR - PD or SD | n=28   - 24 (86%) - 4 (14%) | n=50   - 42 (84%) - 8 (16%) | 0.842 |
| ICD: Irritant contact dermatitis; CR: Complete Response; PR: Partial Response; PD: Progressive disease; SD: Stable disease. | | | |
